# On the Administration of Chloroform

**Published:** 1851-07

**Authors:** John Snow


### ARTICLE XXIX.

### *On the Administration of Chloroform.*

Upon the respective merits of the inhaler and handkerchief, in the administration of chloroform, it is only necessary to enumerate the fatal cases to prove that the handkerchief, and not the use of inhalers, has been the chief cause of the deaths :

I. Jan. 28, 1848.—Hannah Greener, aged 15, near Newcastle ; toe-nail operation; handkerchief.

II. Feb. 23, 1848.—Mrs. Simmons, of Cincinnati, United States ; extraction of teeth ; inhaler, no medical man present.

III. A young woman at Hyderabad, in Hindostan ; distal phalanx of a finger ; handkerchief.

IV. May, 1848.—Mad'lle Stock, aged 30, at Boulogne ; opening an abscess ; handkerchief.

V. Dec., 1848.—Young gentleman at Govan, near Glasgow ; intended toe-nail operation ; handkerchief.

VI. Jan. 24, 1849.—J. Verrier, aged 17, Lyons ; intended amputation of finger ; handkerchief.

VII. Feb. 20, 1849.—Samuel Bennett, Westminster ; amputation of the toe ; handkerchief.

VIII. Aug. 23, 1849.—Mad'lle Labrune, Langres, France ; intended extraction of tooth ; handkerchief.

IX. Oct. 10, 1849.—John Shorter, aged 48, St. Thomas' Hospital ; toe-nail operation ; inhaler, given by a non-medical person.

X. Nov., 1849.—Girl Jones, Shrewsbury ; removal of eyeball ; not stated.

XI. Young lady, Berlin ; intended extraction of tooth ; napkin.

XII. Feb., 1850.—Artilleryman, Mauritius ; amputation of last phalanx of middle finger ; handkerchief.

XIII. Alexander Scott, aged 34, Guy's Hospital ; removal of portion of hand ; handkerchief.

These, I believe, comprise all the recorded cases in which death was clearly due to the administration of chloroform.

In one of the cases it is not stated that a handkerchief was used, though, from the context of the report, that seemed to be the case. Of the other twelve, an inhaler was used in only two of the cases, and in neither of them was it used by a medical man; whilst in the ten instances in which death was caused by chloroform on a handkerchief, it was administered by a qualified medical man in every instance but one. There have been four cases in which death was accidentally caused by chloroform taken by the diseased parties when no one else was present. Two of these deaths occurred in Scotland. A handkerkerchief was used in each instance; and but for the practice of taking chloroform on a handkerchief, these persons would probably never have taken it at all. However, I have not included these cases in the above list. There was a death commonly attributed to chloroform which I have not included in the list—that which occurred at Mr. Robinson's, the dentist; for I believe that that was a case of fatal syncope, due to mental emotion in a subject with fatty degeneration of the heart, and great enlargement of the liver. He was only just beginning to inhale when he suddenly died. In that case an inhaler was employed.

Other supposed causes of the deaths from chloroform have been suggested by various writers, besides those above mentioned, only one of which I shall notice. Many persons, especially in Edinburgh, have attributed the fatal cases to the impurity of the chloroform employed; but, in the first place, it has been examined in nearly all the cases, and found to be quite good. And again, although it might contain impurities which would cause irritation at the time, and subsequent disagreeable effects, it could not become contaminated with any thing likely to cause sudden death; and, before this assertion is repeated, we ought to be told with what substance stronger than itself chloroform is liable to be contaminated. I fully admit the necessity of having chloroform, as well as other medicines, quite pure; as its adulteration, though not liable to cause death, may cause disappointment, and other disagreeable consequences; and it must also be admitted, that this, as well as other drugs, is liable to impurity, both in London and elsewhere. I am of opinion, however, that impure chloroform has not been supplied by the respectable houses of this metropolis. I generally use

Mr. Morson's chloroform, but have, at one time or other, used that of Mr. Squire, Mr. Jacob Bell, Mr. Bullock, Mr. Hooper, Mr. Taylor and others, and always found it good. I have also used the chloroform of Messrs. Duncan, Flockhart & Co., of Edinburgh, and cannot give it any better praise than by saying it was equally good. If the Edinburgh people wish to assert that they can produce something better than pure chloroform, they must call it by another name, for it must be a different medicine.

The real cause of the deaths from chloroform, undoubtedly is, that in each case the patient has had an overdose: I mean more than was necessary to render the patient, or one of similar size and strength, insensible. By a dose of chloroform, however, must be understood the quantity that is in the system at one time, and not the quantity inhaled during an operation. For instance, when the inhalation is left off for two or three minutes, a great part of the chloroform exhales by the breath, and the patient, perhaps, requires to inhale a little more. This should be considered a repetition, and not an increase of the dose.

The necessary points to be observed, in order to avoid the risk of giving an overdose, are—1st, that its vapor be systematically diluted with a sufficient quantity of air, by means of a suitable apparatus, when no accident can happen without the continued neglect of evident warning symptoms; and 2d, that the person administering the chloroform should keep his whole attention directed to the patient, and be able to understand all the signs that occur. I have not space here to go fully into the description of the signs which indicate when the inhalation would cease, and an operation begin, but would rather refer to some papers of mine "on Narcotism by Inhalation," in the Medical Gazette, 2d vol. for 1848, and also to some papers on "Chloroform," by Dr. Sibson, in the same volume. I may state, however, that it is chiefly by attention to the state of the respiration and the eye, that danger is to be avoided. The pulse may be felt, as a physiological inquiry, or with reference to the operation, but gives no guiding information concerning the chloroform, for the following reasons: When the vapor is diluted to a safe extent, it might be continued till death, as I have ascertained in animals, and the pulse would still beat distinctly for many seconds after the respiration had ceased; and if, on the other hand, the vapor be of dangerous strength, the heart might suddenly cease to beat, and the first intimation of danger from the pulse would come only too late.

If chloroform be given on a handkerchief at all, not more than from fifteen to twenty *minims** should be put on at once. In this way, it may be given in midwifery, as that quantity, and often much less, suffices for each application; but in surgery, where the full effect is required, this would be insufficient. —*Dr. John Snow in reply to Prof. Lizars. Dub. Med. Press.*

